# New strains of Japanese encephalitis virus circulating in Shanghai, China after a ten-year hiatus in local mosquito surveillance

**DOI:** 10.1186/s13071-018-3267-9

**Published:** 2019-01-09

**Authors:** Yuan Fang, Yi Zhang, Zheng-Bin Zhou, Shang Xia, Wen-Qi Shi, Jing-Bo Xue, Yuan-Yuan Li, Jia-Tong Wu

**Affiliations:** National Institute of Parasitic Diseases, Chinese Center for Disease Control and Prevention; WHO Collaborating Centre for Tropical Diseases; National Center for International Research on Tropical Diseases, Ministry of Science and Technology; Key Laboratory of Parasite and Vector Biology, Ministry of Health, Shanghai, 20025 People’s Republic of China

**Keywords:** Japanese encephalitis, Mosquito-borne diseases, *Culex tritaeniorhynchus*, *Culex pipiens*, SA14-14-2

## Abstract

**Background:**

Continuous vector pathogen surveillance is essential for preventing outbreaks of mosquito-borne diseases. Several mosquito species acting as vectors of Japanese encephalitis virus (JEV), dengue virus, Zika virus, malaria parasites and other pathogens are primary mosquito species in Shanghai, China. However, few surveys of human pathogenic arboviruses in mosquitoes in Shanghai have been reported in the last ten years. Therefore, in this study, we evaluated mosquito activity in Shanghai, China during 2016 and tested for the presence of alphaviruses, flaviviruses, orthobunyaviruses and several parasitic pathogens.

**Results:**

Five pooled samples were JEV-positive [4/255 pools of *Culex tritaeniorhynchus* and 1/256 pools of *Cx. pipiens* (*s.l*.)] based on analysis of the *NS5* gene. Alphaviruses, orthobunyaviruses, *Plasmodium* and filariasis were not found in this study. Phylogenetic and molecular analyses revealed that the JEV strains belonged to genotype I. Moreover, newly detected Shanghai JEV strains were genetically close to previously isolated Shandong strains responsible for transmission during the 2013 Japanese encephalitis (JE) outbreak in Shandong Province, China but were more distantly related to other Shanghai strains detected in the early 2000s. The E proteins of the newly detected Shanghai JEV strains differed from that in the live attenuated vaccine SA14-14-2-derived strain at six amino residues: E130 (Ile→Val), E222 (Ala→Ser), E327 (Ser→Thr), E366 (Arg→Ser/Pro), E393 (Asn→Ser) and E433 (Val→Ile). However, no differences were observed in key amino acid sites related to antigenicity. Minimum JEV infection rates were 1.01 and 0.65 per 1000 *Cx. tritaeniorhynchus* and *Cx. pipiens* (*s.l*.), respectively.

**Conclusions:**

Five new Shanghai JEV genotype I strains, detected after a ten-year hiatus in local mosquito surveillance, were genetically close to strains involved in the 2013 Shandong JE outbreak. Because JEV is still circulating, vaccination in children should be extensively and continuously promoted. Moreover, JEV mosquito surveillance programmes should document the genotype variation, intensity and distribution of circulating viruses for use in the development and implementation of disease prevention and control strategies.

## Background

Japanese encephalitis virus (JEV) is an arbovirus prevalent throughout Asia and in parts of the western and south Pacific [1, 2]. Similar to other members of the genus *Flavivirus*, JEV is a single-stranded positive-sense RNA virus with an 11-kb genome encoding three structural (capsid, C; pre-membrane, prM; and envelope, E) and seven nonstructural proteins (NS1, NS2A, NS2B, NS3, NS4A, NS4B and NS5) [3, 4]. Rice paddy-breeding *Culex tritaeniorhynchus* is the primary JEV vector [1]. In addition, *Cx. pipiens* [5, 6], *Cx. bitaeniorhynchus* [5], *Cx. modestus* [7] and *Anopheles sinensis* [8] have been shown to transmit JEV in nature. Pigs, wading birds and bats are susceptible reservoirs and act as amplifiers of JEV [9]. Humans, cows and horses are dead-end hosts [10], as they fail to produce viremia at titers sufficient to infect mosquitoes [5]. In nature, JEV is transmitted from vectors to amplifying hosts and then back to vectors, and human outbreaks are the result of spillover effects into the human population [11]. Thus, evaluation of the infection prevalence of JEV in mosquitoes may be important for assessing the risk to public health [12].

An estimated three billion people live in JEV epidemic areas, including China, India and the Southeast Asian peninsula [11]. Approximately 67,900 cases of JEV infection are reported annually [1, 2, 9], although JEV epidemics are highly dynamic [11]. Approximately 50% of cases of JEV infection occur in China [1], potentially because Japanese encephalitis (JE) is a legally notifiable infectious disease in China, whereas effective reporting systems have not been established in most countries with high incidences of JE [6]. In China, JE cases are mainly concentrated in the eastern and southwestern regions, and no local cases have been reported in Xinjiang or Qinghai to date [6]. The principal JEV-susceptible population is children below 15 years of age, and the virus has an incubation period of 5–15 days [13]. Clinically, since the virus can cross the blood brain barrier, JE manifests as a high fever and generalized tonic spasms; thus, in severe cases, irreversible neurologic damage, resulting in consciousness disorders and serious dystonia, may be observed [14, 15].

There is no established treatment for JE [9]. Fortunately, JEV infection is symptomatic in less than 1% of cases [16], and for this reason, JE is often confused with other forms of encephalitis [11]. Thus, the morbidity of JEV is likely to be underestimated [17]. Vaccination programmes, improvements in living standards and sanitation, and the mechanization of agriculture, coincident with economic growth and development, influence the incidence of JE [11]. The most important strategy for preventing JE and reducing the disease burden is increased use of JEV vaccines [16]. In China, the disease burden of JE has declined sharply since the late 1970s, a decade after the introduction of the inactivated vaccine P3 began in economically developed cities, such as Beijing and Shanghai, in 1968 [6]. Subsequently, the vaccine virus strain SA14-14-2 was derived from a wild-type JEV isolated from *Cx. p. pallens* mosquito larvae in Xi’an, China and licensed in 1988 [18]. Owing to its advantages, such as increased efficacy at a lower dose, fewer side effects, and lower cost [13, 19, 20], the attenuated live vaccine SA14-14-2 is now the main vaccine used in China. Moreover, since 2008, the JEV vaccine has been available free of charge to children 0–15 years-old in China, significantly reducing the spread of JEV [21]. Indeed, only 2178 cases were reported in 2013 [22]. However, the proportion of adult JE cases has increased in Henan, Hebei, Shandong, Shanxi, Shaanxi and Gansu Provinces, located north of the Yangtze River in China [6, 21]. An outbreak of JE in 2013 in Shandong Province, China occurred mainly in adults (73% of 407 cases) [12]. Similar phenomena have been observed in other countries in eastern Asia, including Japan [23] and Korea [24].

Japanese encephalitis virus strains originating from the Indonesia-Malaysia region can be divided into five geographical and epidemiological genotypes based on the *E* gene, i.e. GV, GIV, GIII, GII and GI (evolutionary order) [25, 26]. The GI genotype can be further divided into two subgenotypes, i.e. GI-a and GI-b. The distribution of GI-a is restricted to Thailand and Cambodia, while GI-b has been the most commonly isolated JEV genotype this century [27]. The prototype JEV strain (known as Nakayama) was isolated in Japan in 1935 and was recognized to be a member of GIII [28]; this genotype was also identified in China (Beijing-1 virus) in 1949 [6]. GIII was the predominant genotype in temporal JEV epidemic regions from 1935 until the 1980s and was then gradually replaced by GI-b thereafter [6, 27, 29]. JEV GI has been isolated in China since 1979 and has expanded rapidly in endemic areas over the past 35 years, whereas JEV strains isolated before the 1970s generally belonged to GIII [26, 30]. The tendency for GI to gradually replace GIII, becoming the dominant JEV genotype, has been observed in many other regions in Asia and reflects its efficiency in replication [27, 31] and superior tolerance to temperature extremes [32]. However, the host range of GI is more restricted than that of GIII, reflected in the fact that there is greater genetic variation in the *E* gene of GIII than in that of GI [29, 31, 33]. An ancient GV strain was detected in Tibet, China in 2009 [34], 50 years after it was originally identified in a patient in Malaysia in 1952 [35]. Shortly thereafter, another GV isolate was detected in *Cx. bitaeniorhynchus* in Korea in 2010 [36]. Notably, currently available vaccines do not induce appropriate immune protection against GV [37]. The GIV genotype appears to be confined to the Indonesia-Malaysia region, and the latest isolate was collected in the early 1980s [38]. To date, three JEV genotypes, i.e. GI, GIII and GV, have been isolated in China. In contrast, GII was prevalent in Korea in the 1950s and was associated with a JE outbreak among American soldiers during the Korean War [39]. However, GII quickly died off in temperate Asia and is primary sampled from tropical and subtropical regions, such as Malaysia, Indonesia and north Australia [38, 40].

The spread of JEV in Asia originated from Thailand and the Shanghai, Shandong, Sichuan, Yunnan and Zhejiang Provinces of China [41]. In Shanghai, the first JE outbreak on record occurred in 1965 and speculating that the epidemic period was approximately 15 years in Shanghai prior to the implementation of a vaccine programme [42]. By the end of the last century, total positivity rates of the JE H1 antibody in urban and rural residents reached 88.15 and 87.46%, respectively [43]. In 2016, there were two clinical cases in Shanghai reported *via* personal communication with officials at the Shanghai City Disease Prevention and Control. This pattern was also observed in Taiwan Province, China, where routine use of the JEV vaccine began in 1968, and the annual JE incidence is currently between 20 and 40 cases [16]. Thus, JEV is expected to remain a public health issue. However, the most recent JEV genotype identified in Shanghai was available publicly in 2007 [30]. Few surveys of human pathogenic arboviruses in mosquitoes, such as JEV and dengue virus, have been reported in Shanghai in the last ten years.

Shanghai is a high-risk area for arbovirus spread because of the abundance of migratory birds, human migration and international travel [11, 44]. Furthermore, Shanghai has a temperate climate, which may increase the JEV disease burden compared with that in tropical and subtropical regions [45]. Although the implementation of vaccination programmes has dramatically decreased the incidence of JE in Shanghai, epidemic outbreaks, such as incidental JE [5, 12, 29] and dengue [46, 47], have occurred in areas near Shanghai. Notably, the spread of arboviruses is accelerating faster than anticipated [48, 49]. In addition to JEV, imported cases or even local outbreaks of emerging mosquito-borne diseases such as Zika [50, 51], chikungunya [52] and West Nile virus (WNV) [53] have been reported successively in China. Moreover, both malaria and lymphatic filariasis were once prevalent in Shanghai [54, 55]. Although lymphatic filariasis has been eliminated [55] and the local transmission of malaria has been effectively controlled in China [56], the numbers and proportions of imported cases of malaria have continued to increase [57, 58]. Therefore, mosquito-borne pathogen surveillance programmes are urgently needed in Shanghai, an international metropolis with important commercial harbors and nature reserves hosting numerous migratory birds. The primary mosquito species in Shanghai are *Cx. pipiens* (*s.l*.), *Cx. tritaeniorhynchus*, *Aedes albopictus* and *An. sinensis* [59], which act as vectors for multiple human pathogens [60]. It should be mentioned that the *Cx. pipiens* complex encompasses three forms (*pallens*, *molestus*, *quinquefasciatus*) and hybrid forms of *pallens* and *quinquefasciatus* [61]. The complex is widely distributed in China, with intermediates of *Cx. p. pallens* and *Cx. p. quinquefasciatus* found in a zone approximately between 30–32°N [62], within which Shanghai is located. Therefore, *Cx. pipiens* (*s.l*.) here represents all forms of the *Cx. pipiens* complex in Shanghai. It is worth noting that insecticide resistance in mosquito vectors is high in several districts of Shanghai, as for some mosquito vectors that prefer manmade habitats, such as those in irrigated rice fields, larvae are often heavily exposed to pesticide selection pressure [63, 64]. Therefore, in this study, we conducted a survey to determine the presence, genetic variation, geographical distribution and infection rate of JEV and other arboviruses in the area.

## Methods

### Sampling

**Fig. 1:**
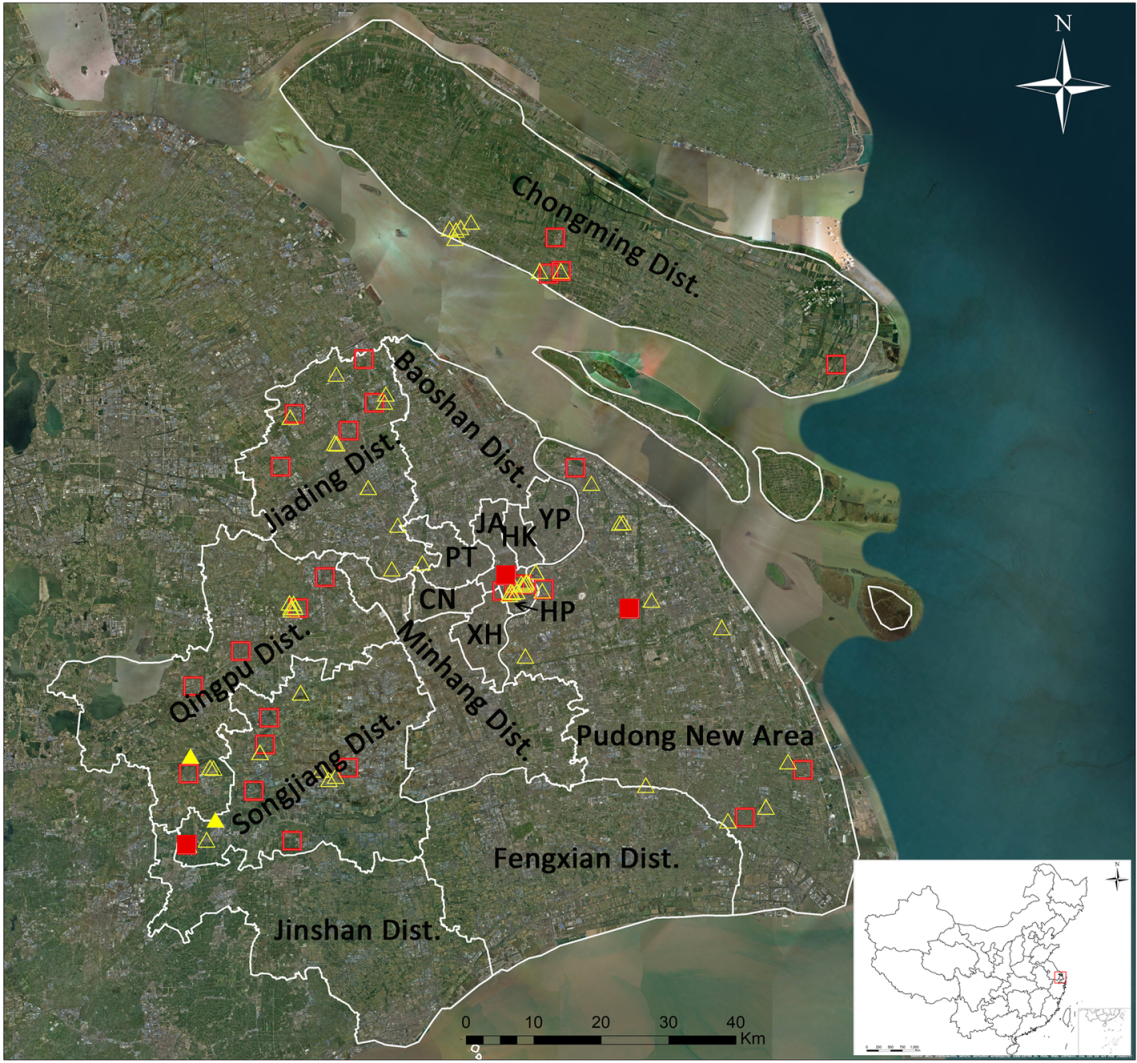
Map of survey sites for the mosquito-borne pathogen surveillance programme in 2016 in Shanghai, China. Squares represent sites using the CO_2_-baited trap, triangles represent sites using the labor hour method and color-filled symbols represent Japanese encephalitis virus detection. *Abbreviations*: HP, Huangpu District; XH, Xuhui District; CN, Changning District; JA, Jing’an District; PT, Putuo District; HK, Hongkou District; YP, Yangpu District

Shanghai is located in the alluvial plain of the Yangtze River Delta, at the mouth of the Changjiang River. In this region, the climate is temperate, which is suitable for mosquito breeding. Arboviral surveillance was carried out from May to November 2016 using CO_2_-baited traps and the labor hour method. CO_2_-baited traps were hung from sunset to sunrise for collecting mosquitoes overnight. The labor hour method was used to catch adult mosquitoes in indoor habitats by using a mosquito aspirator for 15 min within 1 h after sunset. Both methods were performed three times per month, i.e. in the first, second, or third week and in the last week of the month. A total of 94 survey sites covering multiple ecological areas from central, subrural, rural and island regions were examined, as shown in Fig. 1. The map was generated using ArcGIS 10.1 ArcMap software (ESRI, Redlands, CA, USA). Mosquitoes were identified using morphological characteristics according to the national key [61], and blood-containing and male mosquitoes were excluded. Mosquitoes containing blood were excluded to prevent contamination with virus contained in a blood meal. Some morphologically ambiguous specimens were evaluated by molecular methods [65]. Since the vector competence of *Cx. tritaeniorhynchus*, *Cx. pipiens* (*s.l*.), *Ae. albopictus* and *An. sinensis* in transmitting corresponding mosquito-borne pathogens in China has been confirmed [60, 66], we did not dissect each mosquito to separate the salivary gland from the other tissues, but we instead used the entire individual to analyze the presence of target pathogens. Groups of mosquitoes consisting of 1–50 individuals were pooled by species, collection date, method and location; preserved in 75% ethanol; and stored at -20 °C for further virus direct analysis without isolation.

### Nucleic acid extraction and polymerase chain reaction (PCR)

RNA was extracted from pools of *Cx. tritaeniorhynchus*, *Cx. pipiens* (*s.l*.) and *Ae. albopictus* as previously described [67], yielding a final product of 50 μl/pool. First-strand cDNA was synthesized by reverse-transcription PCR (RT-PCR) using a Takara PrimeScript RT Reagent Kit with gDNA Eraser (Takara Bio, Shiga, Japan). To assess the integrity of RNA, the mosquito *18S* gene was amplified using the RT-PCR products [68]. *Flavivirus* was amplified with the primer pair PF1S and PF2R-bis, targeting the partial *NS5* gene (216 bp) [69]. Primer sets JEV-Ef/JEV-Er [70] and prMF/prMR [30] were used to amplify the 1581-nt *E* and 674-nt *prM* genes for further genotype identification. Alphavirus and orthobunyavirus in mosquito samples was amplified by primer sets α6533f/α6999c [71] and BCS82C/BCS332V [72], respectively. PCR products were visualized by 1 or 2% (depending on the length of the amplification fragments) agarose gels with Goldview in 0.5× Tris-acetate-EDTA buffer. Positive products were purified, cloned and sequenced by Sangon (Shanghai, China).

For DNA extraction, ATL (Qiagen, Hilden, Germany) was added to replace the 75% ethanol in pools of *An. sinensis*. Samples were homogenized in a mixer mill (Jingxin, Shanghai, China), with one 5-mm and one 3-mm steel ball added to each tube. The mixture was incubated at 56 °C overnight in an oscillation thermo-block. The samples were then centrifuged at 1000 rpm for 3 min at room temperature. Next, 200 μl of supernatant from each ground sample was added to the Roche® MagNA Pure 96 sample plate, which was placed into to the MagNA Pure 96 System (Roche, Basel, Switzerland) for automated DNA extraction (MagNA Pure 96 DNA and Viral NA Small Volume Kit) according to the manufacturer’s instructions. The extraction process, based on magnetic glass particles, can extract DNA from 96 pools simultaneously in approximately 1 h. After extraction, DNA was eluted in 50 μl of buffer solution. Nested PCR was carried out to detect the presence of five species of *Plasmodium*, including *P. falciparum*, *P. vivax*, *P. malariae*, *P. ovale* and *P. knowlesi*, as described by Yan et al. [73]. In addition, the *SspI* DNA repeat sequence of *Wuchereria bancrofti* [74] and *HhaI* repeat sequence of *Brugia malayi* [75] were detected in the DNA products obtained from pools of *Cx. pipiens* (*s.l*.)and *An. sinensis*, respectively.

### Phylogenetic analysis

Sequences of PCR products were compared with those deposited in the GenBank database using the BLAST program. The obtained flavivirus sequences were aligned with available sequences of the flavivirus *NS5*, *prM* and *E* genes of JEV retrieved from the GenBank database using ClustalW2 [76] with default settings, which were manually adjusted if necessary. Neighbor-joining (NJ) trees were established following Kimura’s two-parameter (K2P) distance model [77] with 1000 bootstrap replicates using MEGA v.7.0 software [78]. Both intra- and inter-genotype *E* gene divergences were examined based on our original obtained sequences and those deposited in GenBank using the K2P distance model in MEGA v.7.0. Based on the Akaike information criterion, the best-fit model for the alignment was determined using Modeltest 3.7, in cooperation with PAUP* v.4.0b10 [79]. Consequently, calculation of the maximum likelihood (ML) and Bayesian likelihood trees was completed under the GTR + I + G model for both the *NS5* and *E* genes, whereas the TrN+G model was used for the *prM* gene. The ML tree was constructed using MEGA v.7.0 software, with 1000 bootstrap replicates. The Bayesian tree was constructed with MrBayes v.3.2.1 [80], run for 10 million generations, with the first 25% of generations discarded as burn-in. The trees were unrooted to provide the least biased topology and visualized using Figtree v.1.4.2 (http://tree.bio.ed.ac.uk/software/figtree/).

### Infection rate calculation

Because the sizes of the pools of collected mosquitoes varied considerably, infection rates were calculated by bias-corrected maximum likelihood estimation (MLE) and minimum infection rate (MIR) using the Excel add-in PooledInfRate v.4 statistical software package [81] and expressed as the number of infected mosquitoes per 1000 individuals.

## Results

### Detection of mosquito-borne pathogens from samples

**Fig. 2:**
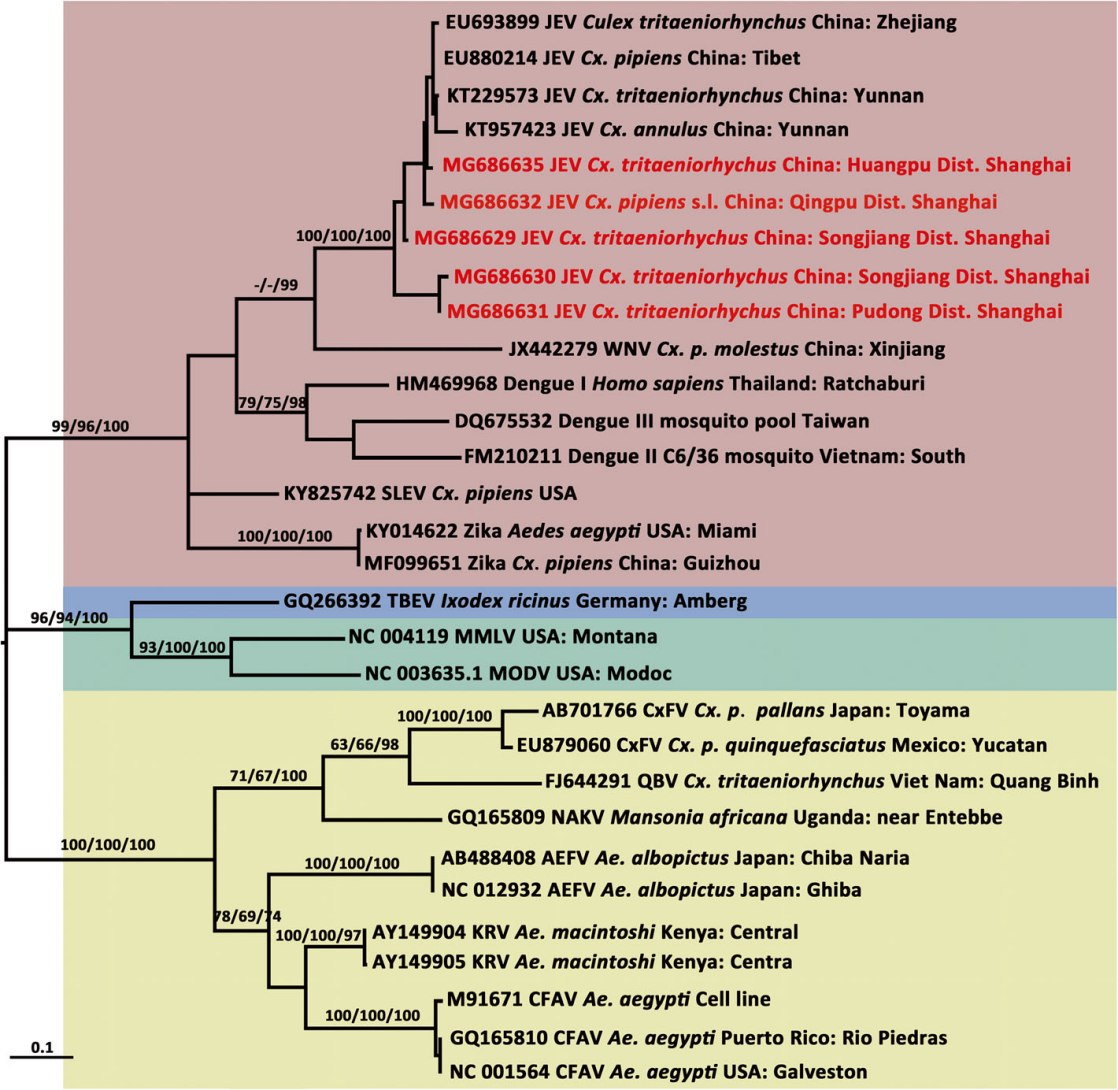
Maximum likelihood phylogenetic tree of partial *NS5* gene sequences of flavivirus. The maximum likelihood tree was constructed by the GTR + I + G model. The GenBank accession number, virus name, origin, and country/province are noted. The JEV sequences obtained in this study are marked in red. The numbers above each branch represent the bootstrap support for the maximum likelihood, neighbor-joining, and Bayesian analyses, respectively, based on 1000 replicates. The scale-bar indicates 0.1 substitutions per site. Sequences shaded tan represent mosquito-borne flavivirus, those shaded sky blue represent tick-borne flavivirus, those shaded aquamarine represent no-known vector flavivirus, and those shaded khaki represent insect-specific flavivirus. *Abbreviations*: JEV, Japanese encephalitis virus; SLEV, Santa Louis encephalitis virus; TBEV, tick-borne encephalitis virus; MMLV, Montana myotis leukoencephalitis virus; MODV, Modoc virus; CxFV, Culex flavivirus; QBV, Quang Binh flavivirus; NAKV, Nakiwogo virus; AEFV, Aedes flavivirus; KRV, Kamiti River virus; CFAV, cell fusing agent virus

**Fig. 3:**
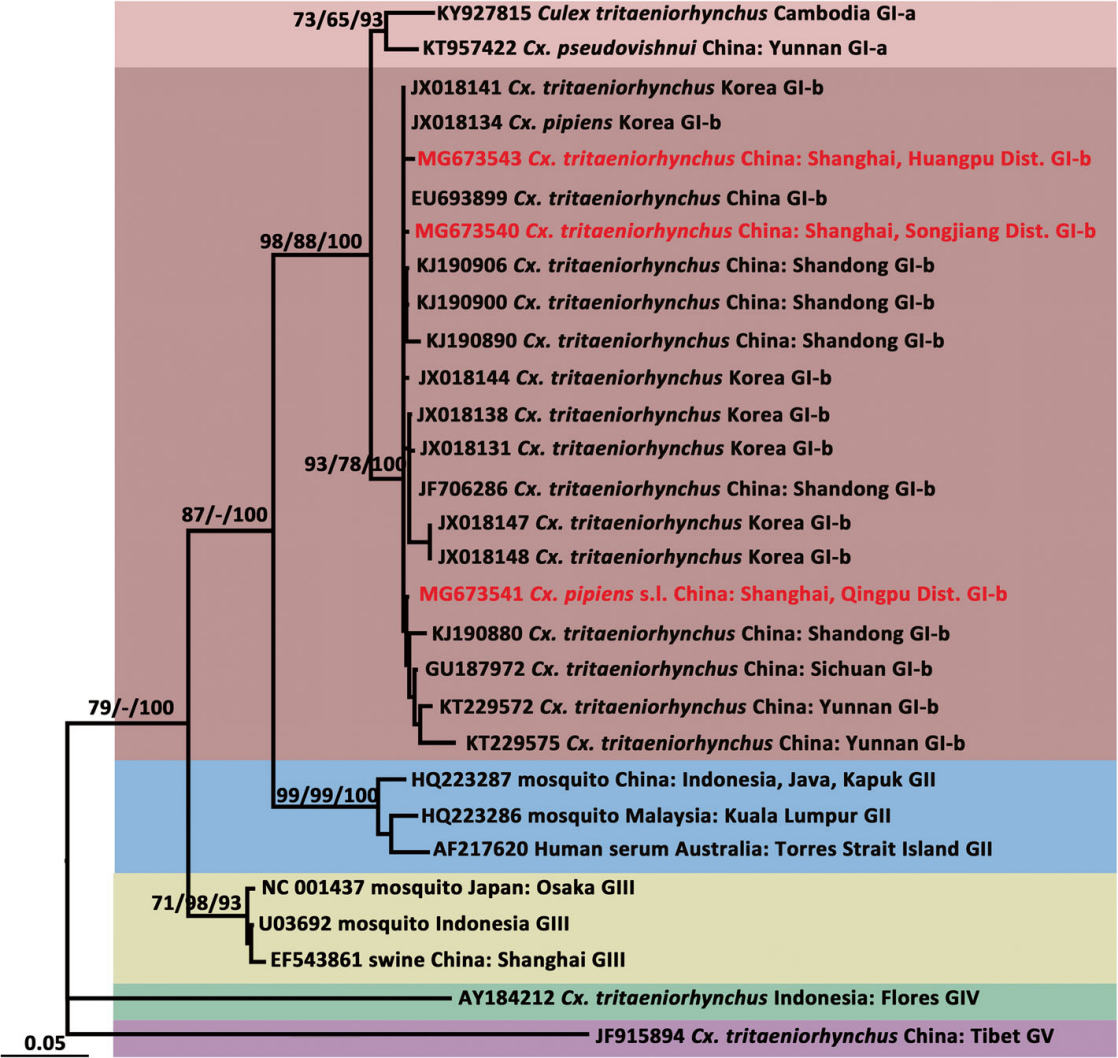
Maximum likelihood phylogenetic analysis of Japanese encephalitis virus pre-membrane gene sequences. The maximum likelihood tree was constructed under the TrN + G model. The GenBank accession number, origin, country/province and genotype of each strain are noted. The JEV sequences obtained in this study are marked in red. The numbers above each branch represent the bootstrap support of the maximum likelihood, neighbor-joining, and Bayesian analyses, respectively, based on 1000 replicates. The scale-bar indicates 0.05 substitutions per site. Sequences shaded tan represent the GI-a genotype, those shaded rose-brown represent the GI-b genotype, those shaded sky blue represent the GII genotype, those shaded khaki represent the GIII genotype, those shaded aquamarine represent the GIV genotype, and those shaded thistle represent the GV genotype

**Fig. 4:**
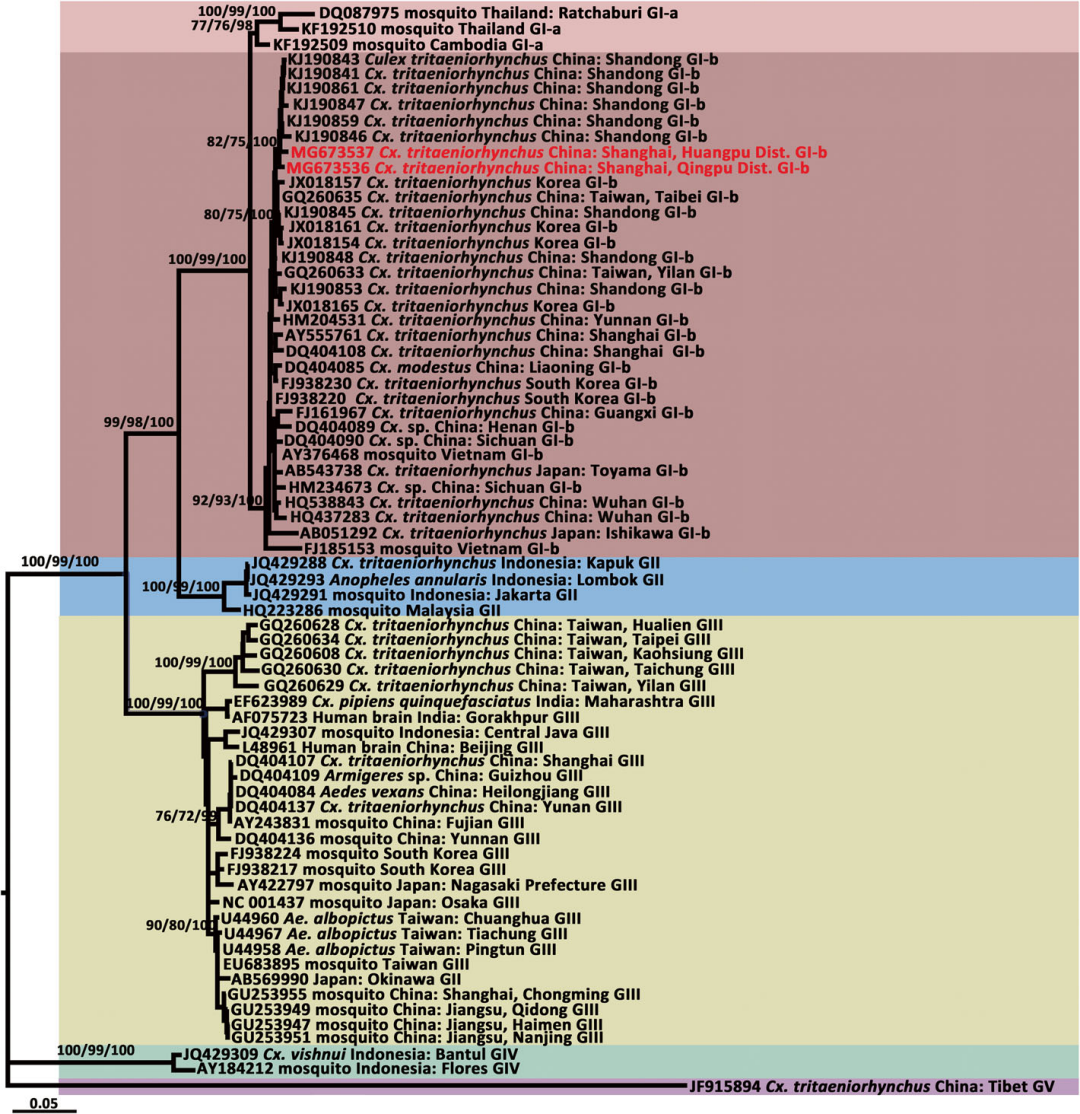
Maximum likelihood phylogenetic analysis of Japanese encephalitis virus envelope gene sequences. The maximum likelihood tree was constructed under the GTR + I + G model. The GenBank accession number, origin, country/province and genotype of each strain are noted. The JEV sequences obtained in this study are marked in red. The numbers above each branch represent the bootstrap support of the maximum likelihood, neighbor-joining, and Bayesian analyses, respectively, based on 1000 replicates. The scale-bar indicates 0.05 substitutions per site. Sequences shaded tan represent the GI-a genotype, those shaded rose-brown represent the GI-b genotype, those shaded sky blue represent the GII genotype, those shaded khaki represent the GIII genotype, those shaded aquamarine represent the GIV genotype, and those shaded thistle represent the GV genotype

In total, 21,881 adult mosquitoes belonging to nine species from five genera of the family Culicidae (*Culex*, *Aedes*, *Anopheles*, *Mansonia* and *Coquillettidia*) were collected at 94 survey sites (Fig. 1) during the peak mosquito activity period from May to November 2016 in Shanghai. Among them, 10,004 (45.72%) were *Cx. tritaeniorhynchus*, 4813 (22.00%) were *An. sinensis*, 3385 (15.47%) were *Cx. pipiens* (*s.l*.), 3365 (15.38%) were *Ae. albopictus*, 150 (0.69%) were *Cx. inatomii*, 111 (0.51%) were *Ae. dorsalis*, 32 (0.15%) were *Ae. vexans*, 20 (0.09%) were *Mansonia uniformis* and one (0.01%) was *Coquillettidia ochracea*. The geographical distribution of the survey sites is shown in Fig. 1. Among the collected mosquitoes, sites located on Chongming Island had the highest diversity of mosquito species, particularly sites closest to the Chongming Dongtan National Nature Reserve. For arbovirus detection by RT-PCR, 255 of 655 pools of *Cx. tritaeniorhynchus*, 256 of 611 pools of *Cx. pipiens* (*s.l*.) and 257 of 456 pools of *Ae. albopictus* were randomly chosen. Moreover, 192 of 362 pools of *An. sinensis* were randomly chosen for DNA extraction for further parasitic pathogen detection. According to sequence identity and phylogenetic analysis (Fig. 2), five pools were positive for the JEV *NS5* gene, including four pools of *Cx. tritaeniorhynchus* (two pools from Songjiang District, one pool from Huangpu District and one pool from the Pudong New Area) and one of *Cx. pipiens* (*s.l*.) (from Qingpu District). The *prM* and *E* genes were successfully amplified in three and two of the five JEV-positive pools, respectively. Phylogenetic analyses (Figs. 3, 4) indicated that GI was the only genotype detected among samples collected in Shanghai in 2016. Collection information, host species and GenBank accession numbers are shown in Table 1. No sequences ascribable to the PCR target were obtained using the screening PCR for alphaviral and orthobunyaviral genomes. Moreover, no parasites, including *Plasmodium* spp., *W. bancrofti* and *B. malayi*, were found in this study.

**Table 1:** Details of Japanese encephalitis virus strains detected from culicines, captured in Shanghai during May to November 2016

Strain	Host	Collection date	Geographical location	Habitat	GenBank ID		
					*NS5*	*E*	*prM*
PD3G_16-9-S-Cut-R-10-3	*Culex tritaeniorhynchus*	23-Sep-16	Songjiang District	Livestock farm	MG686629		MG673540
PD3H_16-9-S-Cut-C-4-1	*Cx. tritaeniorhynchus*	2-Sep-16	Songjiang District	Suburb residential area	MG686630		
PD8F_16-9E-P-Cut-C-2-21	*Cx. tritaeniorhynchus*	2-Sep-16	Pudong New Area	Suburb residential area	MG686631		
QP5E_16-7L-Q-Cup-R-4-1	*Cx. pipiens* (*s.l.*)	24-Jul-16	Qingpu District	Suburb residential area	MG686632	MG673536	MG673541
HP4A_16-7-H-Cut-C-5-2	*Cx. tritaeniorhynchus*	22-Jul-16	Huangpu District	Urban residential area	MG686635	MG673537	MG673543

*Abbreviations*: *NS5* non-structural 5 gene, *E* envelope gene, *prM* pre-membrane gene

### Molecular characterization and phylogenetic analysis based on the *prM* and *E* genes of JEV

The phylogenetic tree based on the *NS5* gene (Fig. 2) showed that the genus of *Flavivirus* contains four distinguishable clusters, including mosquito-borne flavivirus, tick-borne flavivirus, no-known vector flavivirus and insect-specific flavivirus. All five JEV-positive sequences (MG686629, MG686630, MG686631, MG686632 and MG686635) clustered within the JEV clade. WNV, spread by *Culex*, was found to be genetically close to JEV.

The topologies of the trees produced from the JEV *prM* (Fig. 3) and *E* genes (Fig. 4) identified five major clades, including genotypes I, II, III, IV and V. Moreover, GI was composed of two distinct clades, representing the two sub-genotypes, GI-a and GI-b. Based on the supporting values of the three phylogenetic trees (Figs. 3, 4), for the majority of lineages, the Bayesian method returned relatively higher bootstrap values than the ML and NJ methods. The *prM* (650 nt) tree showed some lower bootstrap values but displayed a topology consistent with that obtained with the *E* gene sequence (1500 nt).

The sequences of the JEV *prM* gene from the Songjiang (PD3G_16-9-S-Cut-R-10-3, MG673540), Qingpu (QP5E_16-7L-Q-Cup-R-4-1, MG673541) and Huangpu (HP4A_16-7-H-Cut-C-5-2, MG673543) strains showed high levels of identity with each other at the nucleotide (range: 99.08–99.38%) and amino acid (range: 99.34–100%) levels, but lower homology to the SA14-14-2 strain (89.09–89.40% at the nucleotide level and 96.05–97.71% at the amino acid level). In the phylogenetic tree based on the *prM* gene (Fig. 3), the Qingpu, Huangpu and Songjiang strains formed a cluster with other GI-b sequences. The Huangpu and Songjiang strains were genetically similar but were relative more distantly related to the Qingpu strain, which was detected in *Cx. pipiens* (*s.l*.).

According to sequence homology analyses based on the *E* gene, the Qingpu strain (MG673536) shared 87.99% nucleotide identity and 97.00% amino acid identity with that of the live attenuated vaccine SA14-14-2, while the Huangpu strain (MG673537) shared 87.58% nucleotide and 96.60% amino acid identity with SA14-14-2. As expected, 99.00% similarity was observed between the Qingpu and Huangpu strains, both at the nucleotide and amino acid levels. In the phylogenetic tree based on the *E* gene (Fig. 4), these strains fell into the GI-b cluster and were most closely related to the Shandong strains, which were suspected to have contributed to the JE outbreak in Shandong Province, China in 2013 [12]; however, they were more distantly related to previously detected local strains, including the SH101 (AY555761, in 2001) and SH05-24 strains (DQ404108, in 2005), both isolated from *Cx. tritaeniorhynchus*.

**Fig. 5:**
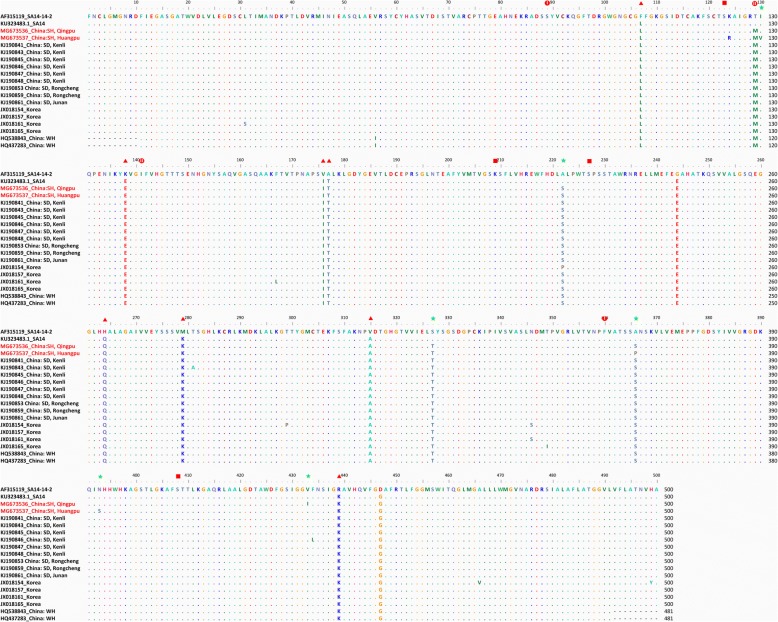
Sequence comparison of amino acid differences in the envelope protein of Japanese encephalitis virus (JEV). Sequence comparisons were performed among the live attenuated vaccine SA14-14-2, SA14, and newly detected Shanghai JEV strains (marked in red), as well as strains suspected to have contributed to prior Japanese encephalitis outbreaks (2013 in Shandong Province, China; 2010 in Korea; and 2006 in Wuhan Province, China) near Shanghai. Dots indicate consensus. The GenBank accession numbers and countries/provinces are noted. Triangles represent eight amino acids (F107L, K138E, V176I, A177T, H264Q, M279K, V315A, and R439K) related to virus attenuation; circles represent two pairs of co-evolving sites (residues S89N to F360Y and M129T to I141V) observed in GI; squares represent four sites (E123, E209, E327, and E408) in the E protein used for haplotype definitions; stars represent the amino acids (I130V, A222S, S327T, R366S/P, N393S, and V433I) of newly detected Shanghai JEV strains that were not consistent with those in the SA14 strain

Deduced amino acid differences in E protein sequences were aligned for comparison among Huangpu and Qingpu strains, strains involved in JE outbreaks, and SA and vaccine strains currently used in China (Fig. 5). Six amino acid residues in the newly detected Shanghai JEV strains differed from those in the live attenuated vaccine SA14-14-2-derived strain (SA14): E130 (Ile→Val), E222 (Ala→Ser), E327 (Gly→Glu), E366 (Arg→Ser/Pro), E393 (Asn→Ser) and E433 (Val→Ile).

**Fig. 6:**
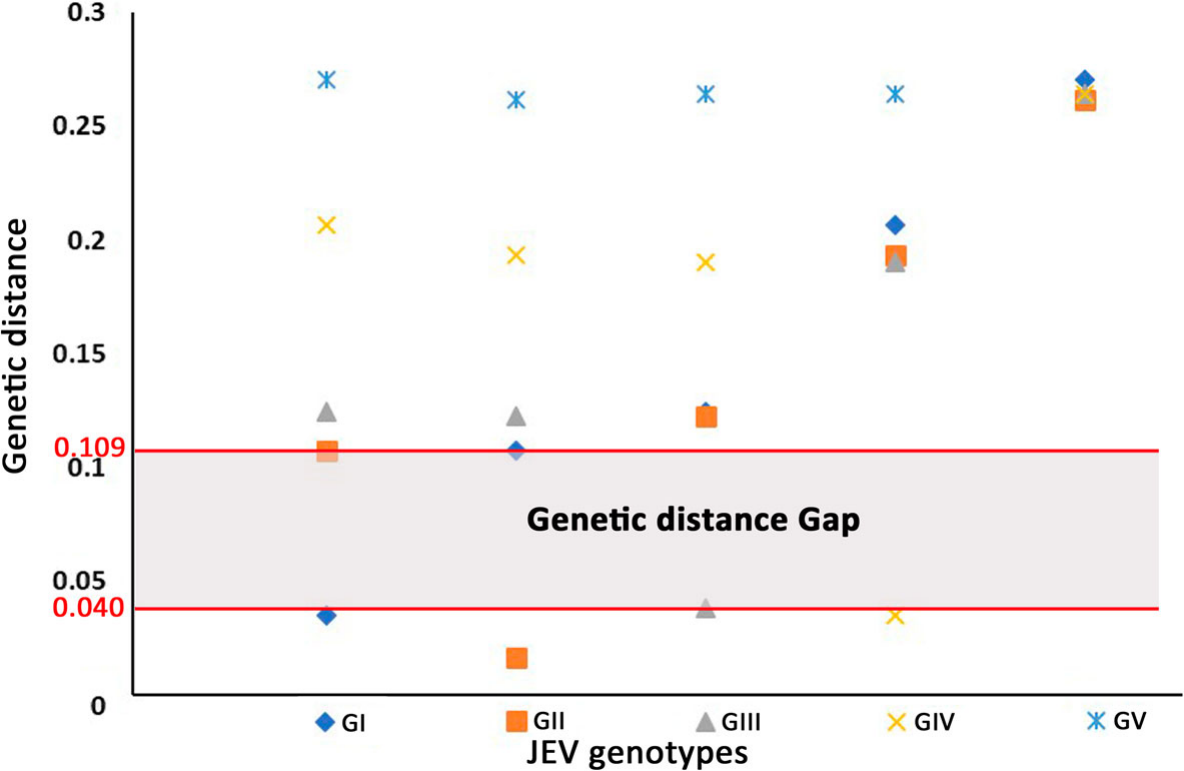
Genetic distances of the envelope genes from five genotypes of Japanese encephalitis virus (JEV). Genetic distance analyses were performed using Kimura’s two-parameter model based on 69 JEV envelope sequences from GenBank and two sequences obtained in this study. The GenBank accession numbers of involved sequences are available in Fig. 4. Y-axis, genetic divergence; X-axis, JEV genotypes

Genetic distance analyses of the *E* gene based on 69 sequences from GenBank and the two obtained here (GenBank accession numbers available in Fig. 4) showed that the average K2P distances within and between JEV genotypes were 0.032 (range: 0.018–0.039) and 0.202 (range: 0.109–0.272), respectively (Fig. 6). On average, the differences between genotypes were 6-fold higher than those within genotypes. The maximum K2P distance within genotypes was observed in GIII (0.039), and the minimum K2P distance between JEV genotypes was 0.109. Thus, the genetic distances among JEV genotypes ranged from 0.039 to 0.109.

### Infection rate of JEV in culicines

The infection rate (Table 2) according to bias-corrected MLE and MIR of JEV in *Cx. tritaeniorhynchus*, both with 95% confidence intervals (CI), were 1.01 (0.33–2.42) and 1.01 (0.02–2.01) per 1000, respectively. Those of JEV in *Cx. pipiens* (*s.l.*) were 1.01 (0.00–1.92) and 0.65 (0.04–3.14) per 1000, respectively. The overall bias-corrected MLE infection rates and MIR of JEV in culicines were 0.91 (0.34–2.01) and 0.91 (0.11–1.71) per 1000 vectors, respectively. Four out of five positive pools originated from residential areas; three were collected in suburbs, whereas one was collected in the central city region (Huangpu District). The remaining pool was collected from a livestock farm.

**Table 2:** Bias-corrected maximum likelihood estimation (MLE) and minimum infection rate (MIR) of Japanese encephalitis virus in Shanghai, China from May to November 2016

Host	No. of individuals	No. of PP	No. of pools	Positive pool rate (%)	MLE (95% CI)	MIR (95% CI)
*Culex tritaeniorhynchus*	3945	4	255	1.57	1.01 (0.33–2.42)	1.01 (0.02–2.01)
*Cx. pipiens* (*s.l*.)	1540	1	256	0.39	1.01 (0.00–1.92)	0.65 (0.04–3.14)
Overall	5485	5	511	0.98	0.91 (0.34–2.01)	0.91 (0.11–1.71)

*Abbreviations*: *PP* positive pool, *CI* confidence interval

## Discussion

### Historical co-circulation of JEV GI and GIII strains in Shanghai

The first JEV strain found in Shanghai was isolated from a human brain in 1987 and classified as the GIII genotype [30]. In contrast, GI was first detected from *Cx. tritaeniorhynchus* in Shanghai in 2001 [82]. GI and GIII were found alternately between 2003 and 2008, suggesting that these two genotypes co-circulated in this area, although they were never identified in the same year [30]. Thereafter, JEV mosquito surveillance and arbovirus detection in mosquitoes have seldom been carried out in Shanghai. To the best of our knowledge, no data on JEV genotypes has been reported within the last ten years. However, although the implementation of vaccination programmes has dramatically decreased the incidence of JE in Shanghai, JEV will probably continue to circulate in nature based on the existence of annual JE cases.

### A single genotype (GI) has been detected in Shanghai after a ten-year hiatus in JEV mosquito surveillance

In this study, we carried out mosquito-borne surveillance from May to November 2016. The predominant species of mosquitoes collected were *Cx. tritaeniorhynchus*, *Cx. pipiens* (*s.l*.), *Ae. albopictus* and *An. sinensis*. Here, *Cx. tritaeniorhynchus* yielded the majority of JEV-positive pools, indicating that *Cx. tritaeniorhynchus* is the primary vector of JEV, whereas *Cx. pipiens* (*s.l*.) may play some role in JEV circulation in Shanghai. GI was previously thought to be almost exclusively restricted to *Cx. tritaeniorhynchus* [33]. However, in this study, this genotype was also detected in *Cx. pipiens* (*s.l*.). This is the first record of JEV being detected in *Cx. pipiens* (*s.l*.) in Shanghai. *Cx. pipiens* (*s.l*.) and is found mostly in small waste water reservoirs surrounding houses in urban areas in which drainage and sanitation are inadequate, whereas *Cx. tritaeniorhynchus* is mainly distributed in rural and sub-rural areas. Thus, the JEV transmitted by *Cx. pipiens* (*s.l*.) may be more harmful to public health.

In this study, a single genotype of JEV (GI) was detected in Shanghai, which is not surprising given that GIII has not been detected in some areas of Asia for several years. In Korea, GIII was not isolated from 1995 to 2010, although both GI and GIII were detected in Korea in 1994 [5]. Additionally, mosquito pathogen detection results have suggested that only JEV GI strains were involved in the 2010 outbreak [5]. Even in Vietnam, GI was the only JEV genotype detected after 2004 [83, 84]. Furthermore, a low genetic diversity (99.00% identity at both the nucleotide and amino acid levels) of the *E* gene was observed between newly detected Shanghai JEV strains, suggesting that frequent JEV transmission occurs in local areas. However, these strains are genetically distant from a previously detected Shanghai strain (SH101 strain in 2001, AY555761) according to *E* gene sequences, sharing only 87.3% and 96.6% identity at the nucleotide and amino acid levels, respectively. These findings suggest that the haplotype of GI circulating in local areas has changed, likely *via* mutation or introduction from northeastern areas of Asia, such as Shandong Province or Korea, as implied by the close genetic relationships among these strains.

### Sequence comparison with strains involved in JE outbreaks and vaccine strain currently used in China

Both the inactivated P3 vaccine and the live attenuated SA14-14-2 vaccine are derived from GIII strains [6]. The vaccines appear to be effective against four genotypes (GI–GIV) of the virus [9, 16, 19]. In case-control studies in Sichuan Province, China, a single dose of the live attenuated SA14-14-2 vaccine was found to be 80% effective (95% CI: 44–93%), whereas administration of two doses increased the efficacy to 97.5% (95% CI: 86–99.6%) [85]. The effectiveness of a single dose of the SA14-14-2 vaccine varied with regard to the duration of protection, the chance of exposure to wild JEV, and sampling bias [86, 87].

In contrast, a recent study showed that the vaccine SA14-14-2 failed to induce appropriate immune protection against GV [37]. According to sequence homology analyses of the E protein, the recurrent genotype GV (JF915894) was found to have a significantly low identity (79.30%) with the live attenuated vaccine SA14-14-2 at the amino acid level. The E protein is the major constituent of the mature virion surface and is under continuous selection pressure owing to its roles in infectivity and immunity processes, including hemagglutination, virus neutralization and viral particle assembly [6, 27]. The *E* gene has been shown to provide reliable information reflecting the broad geographical and temporal relationships of JEV [32]. Additionally, a small number of mutations in the E amino acid sequence have been shown to be associated with host adaptation and influence the efficiency of mosquito oral infectivity [25]. Eight amino acids (F107L, K138E, V176I, A177T, H264Q, M279K, V315A and R439K) are related to virus attenuation, and residue E138 is particularly important in this process [18]. The co-evolution of two pairs of sites (residues S89N to F360Y and M129T to I141V) was observed in GI, in which these residues functionally interact with each other to maintain a functional E protein [27]. Moreover, 12 haplotypes were defined based on the four sites in the E protein (E123, E209, E227 and E408) predicted to be under positive selection, with SKSS as the predominant haplotype [33]. Thus, we compared the amino acid residues of the live attenuated vaccine SA14-14-2 with the SA14, Qingpu and Huangpu strains, as well as strains involved in recent JE outbreaks [5, 12, 88] near Shanghai. However, there were no corresponding mutations at the E107, E138, E176, E177, E264, E279, E315 or E439 loci. Although mutations in residue 129 occurred in wild JEV strains compared with the SA14 strain sequence, no corresponding paired mutations were identified in E141. All of the observed JEV strains carried the dominant haplotype, SKSS. Notably however, six amino acid residues (I130V, A222S, S327T, R366S/P, N393S and V433I) in the newly detected Shanghai JEV strains differed from the vaccine-derived strain. Thus, continuous surveillance of JEV should be sustained to better understand the genetic characteristics of circulating JEV and to avoid potential breakthrough infection (after vaccination), caused by amino acid mutation at key loci related to antigenicity. Additionally, it has been reported that GI has rarely been isolated from human serum and replicates much more effectively in mosquito and porcine cell lines than in human cell lines, as compared with the performance of GIII [31]. However, all cases of JEV in the last ten years in most parts of Asia have been due to GI [5, 12, 29, 88, 89]. This indicates that GI strains may have evolved to be more effective at infecting humans or are the result of genotype shift in vectors and amplifying hosts. These findings raise concerns regarding potential changes in the epidemiological characteristics of the virus and the effectiveness of vaccines [29]. In addition, the development of novel vaccines is necessary to prevent the recurrence of GV in Asia.

### Extensive promotion of vaccinations and sustained JEV mosquito surveillance are crucial for JE prevention and control in China

Since 2010, the annual number of JEV cases in China has decreased to approximately 2000 [22]. However, JE is still prevalent in Yunnan, Guizhou and Guangxi Provinces, particularly in very remote areas where the administration of JEV vaccinations is not as common [6]. Moreover, in these rural areas, livestock may shed viruses near humans, and breeding habitats, such as paddy fields, may be nearby. These conditions facilitate JEV circulation and amplification, resulting in spillover of the virus into the human population and triggering epidemics. Accordingly, these findings suggest that all domestic pigs should be moved to communal piggeries several kilometers away from the homes of humans in order to reduce the risk of JEV transmission to humans [10]. However, the most effective approach for controlling JEV is thought to be full coverage vaccination of susceptible populations (i.e. children ages 0–15 years) [9]. It is also possible that JE outbreaks may occur in some areas of eastern and middle Asia, where the incidence of JE has been reduced to low levels for several years while JEV continues to circulate in the field, as has been observed for the recurrence of JEV epidemics in Wuhan Province [88]. Thus, areas with a few or no reported JE cases but historical epidemics should be subjected to periodic vector surveillance to monitor the dynamics of JEV prevalence in local areas. More importantly, it is necessary to implement immunization programmes for children in these areas. Additionally, emergency vaccination should be conducted in adults with no history of JEV vaccination during JE outbreaks or when the local JEV infection rate in vectors reaches the level of an epidemic [21].

The infection rate of JEV in Shanghai was found to be 1.01 per 1000 *Cx. tritaeniorhynchus*. The actual infection rate in the field is probably underestimated because samples in the present study were directly tested by RT-PCR, without virus amplification or isolation, since the mosquitoes were preserved in 75% ethanol. Compared with those during JE outbreaks in Shandong Province, China [12] and Korea [5], which averaged 9.1 and 11.8 per 1000 vectors, respectively, the infection rate of JEV in Shanghai in 2016 was ten times lower. However, the infectious prevalence of flavivirus, e.g. WNV (data unavailable for JEV), that represents an “epidemic risk” is more than 5 per 1000 mosquitoes [12]. Thus, the infection rate of JEV in Shanghai derived here is on the same order of magnitude as the epidemic risk of WNV, indicating that it is crucial to promote continuous mosquito-borne virus surveillance in Shanghai.

## Conclusions

In summary, our findings showed that four out of five JEV-positive pools were collected from residential areas, suggesting an increased risk of human infection in Shanghai. Thus, it is necessary to promote continuous, full coverage vaccination in children, supplemented with surveillance of the virus carrier rate of mosquito vectors. Our results also highlight the importance of documenting the genotype distributions and genetic variations in JEV over time in order to establish strategies for the control of mosquitoes and mosquito-borne diseases. Further studies are also needed to evaluate the potential of pigs to act as reservoirs and to determine the role of bird migration in JEV spread.

## Abbreviations

CI: Confidence interval
*E* gene: Envelope gene
JE: Japanese encephalitis
JEV: Japanese encephalitis virus
K2P: Kimura’s 2-parameter
MIR: Minimum infection rate
ML: Maximum likelihood
MLE: Maximal likelihood estimation
NJ: Neighbor-joining
*NS5* gene: Nonstructural5 gene
*prM* gene: Pre-membrane gene
WNV: West Nile virus
